# 2',3'‐Cyclic‐nucleotide 3'‐phosphodiesterase contributes to epithelial‐mesenchymal transition of lens epithelial cells through the notch signalling pathway

**DOI:** 10.1111/cpr.12707

**Published:** 2019-10-16

**Authors:** Yue Li, Yu Zhao, Yan Wang

**Affiliations:** ^1^ Clinical College of Ophthalmology Tianjin Medical University Tianjin China; ^2^ Tianjin Key Laboratory of Ophthalmology and Visual Science Tianjin Eye Institute Tianjin Eye Hospital Tianjin China; ^3^ Technology Transfer Center Kunming Medical University Kunming China

**Keywords:** 2',3'‐cyclic‐nucleotide 3'‐phosphodiesterase, anterior subcapsular cataracts, epithelial‐mesenchymal transition, fibrosis, lens epithelial cell, notch signalling pathway

## Abstract

**Objectives:**

Fibrosis is a complex process involved in multiple diseases that result in organ injury and failure. Cataract, one common form of ocular fibrosis, is a main cause of blindness worldwide, and surgery may be the only cure. In this regard, epithelial‐mesenchymal transition (EMT) of lens epithelial cells (LECs) is the primary cause of anterior subcapsular cataract (ASC). This study aimed to investigate the mechanism by which 2',3'‐cyclic‐nucleotide 3'‐phosphodiesterase (CNPase) regulates the function of EMT in LECs.

**Materials and Methods:**

A mouse model of ASC was used to observe the expression of CNPase in the lens and correlate its expression changes with lens EMT. Furthermore, the effects of CNPase on cell migration and cell proliferation were evaluated by transwell migration, wound healing and EdU staining assays. Finally, Western blotting and immunofluorescence were used to assess the mechanical properties potentially involved in the regulation of EMT by CNPase.

**Results:**

The expression of CNPase was upregulated in LECs during the EMT process in mice with ASC. Notably, CNPase significantly promoted the proliferation, migration and EMT of LECs in vitro. Interestingly, the EMT‐promoting mechanism of CNPase may be achieved by targeting the Notch signalling pathway.

**Conclusions:**

Considering the involvement of EMT in ASC, both CNPase and the Notch signalling pathway may be therapeutic targets for the treatment of cataracts.

## 1 INTRODUCTION

Fibrosis is considered a common cause of organ dysfunction and failure, and the survival rate of several types of fibrosis is even lower than that of cancer.^1^, ^2^ Although fibrosis may involve multiple organs, as in hepatic fibrosis, renal fibrosis, pulmonary fibrosis and ocular fibrosis, there are still some common underlying mechanisms. Accumulating evidence suggests that epithelial‐mesenchymal transition (EMT) is a major contributor to the progression of organ fibrosis.^3^ During the process of EMT, epithelial cells secrete excessive extracellular matrix components, including collagen type I (Col I) and fibronectin (FN) and lose apical‐basal polarity through degradation of the transmembrane protein E‐cadherin,^4^, ^5^ leading to the loss of cell‐to‐cell adhesion and, ultimately, the loss of epithelial cell morphology. Moreover, the cells synthesize α‐smooth muscle actin (α‐SMA) and vimentin, which are typically upregulated in cells with mesenchymal phenotypes.^6^, ^7^

Extensive studies have suggested that the lens serves an ideal biological tool for exploring the mechanism of fibrosis and EMT because of its unique biological properties.^8^ Lens fibrosis is the common mechanism underlying most cataracts, which are responsible for a large proportion of blindness worldwide. Cataract is a common age‐related disease characterized by a progressive increase in lens opacity and light obstruction and a gradual loss of vision.^9^, ^10^ It has been documented that 96% of the population over 60 years of age have varying degrees of lens opacity. With the ageing of the world's population, the incidence of cataracts is increasing rapidly.^11^, ^12^ Cataracts are basically divided into two types by the location of fibrosis: anterior subcapsular cataract (ASC) and posterior capsular opacification (PCO). The proliferation and migration of lens epithelial cells (LECs) to the acellular posterior capsule and their ultimate conversion to myofibroblast‐like cells, known as EMT, are the common causes of ASC and PCO. TGF‐β is considered the pivotal factor in inducing EMT during development, cancer and other pathological conditions. In some epithelial cell lines cultured in vitro, EMT can be induced by TGF‐β stimulation alone; furthermore, EMT is mediated by the Notch, Wnt/β‐catenin and integrin signalling pathways.^13^, ^14^ Therefore, the regulation of EMT has been considered a therapeutic strategy to prevent the occurrence of ASC and PCO and to reduce the incidence of postoperative complications.^7^, ^15^, ^16^

Abnormal Notch signalling has been found in a variety of fibrotic diseases.^16^, ^17^ Inactivation of Notch signalling plays a key role in reversing the EMT phenotype, improving symptoms and overall survival in patients with fibrosis.^17^ It has currently been established that there are four cell surface transmembrane receptors (Notch 1‐4) in mammals. The Notch receptor is cleaved by γ‐secretase, releasing the Notch intracellular domain (NICD), which is then transferred to the nucleus to regulate downstream transcription factors, including Hes and Hey.^18^, ^19^ Previous studies have suggested that Notch signalling interacts with multiple signalling pathways, in which adenosine induces Notch signalling through corresponding receptors, suggesting that the specific regulation of adenosine metabolism may treat fibrotic diseases through the Notch pathway.

2',3'‐Cyclic‐nucleotide 3'‐phosphodiesterase (CNPase) is a key enzyme involved in the process of adenosine metabolism that catalyses the hydrolysis of 2’,3’‐cyclic nucleotides to the corresponding 2'‐adenosine monophosphate (AMP). These AMPs are then converted to adenosine.^20^ Studies have shown that traumatic brain injury triggers the abnormal expression of CNPase, which is related to an increase in AMP and adenosine in the cerebrospinal fluid (CSF). Activation of the mitochondrial permeability transition pore (MPTP) by AMP is hypothesized to lead to apoptosis and necrosis.^21^, ^22^ In the brain, a lack of CNPase leads to increased susceptibility to brain damage and neurological diseases.^23^ Surprisingly, the lack of CNPase in the kidney protects against kidney damage, possibly by preventing the formation of 2'‐AMP and reducing mitochondrial damage.^24^, ^25^ Therefore, the exact roles of 2',3'‐cNMP and its downstream metabolites remain to be fully elucidated.

In this study, we focused on the role of CNPase in lens epithelial cell EMT. We used a mouse ASC model to observe the expression of CNPase in the lens and correlate its expression changes with lens EMT. Here, we show a possibly unreported mechanism of CNPase that regulates the function of EMT by directly targeting Notch signalling. These results suggest that CNPase and the drug‐targeted Notch pathway may hold promise in the design of therapeutic strategies for preventing and treating ASC.

## 2 MATERIALS AND METHODS

### 2.1 Cell culture

The human lens epithelial cell line SRA 01/04 was purchased from Bena Culture Collection. The cells were grown in Dulbecco's Modified Eagle Medium (DMEM) (Gibco; Thermo Fisher Scientific, No.: 12100046) containing 10% foetal bovine serum (FBS, Gibco; Thermo Fisher Scientific, No.: A3161002C) and 1% penicillin/streptomycin. Cell cultures were maintained at 37°C in a humidified 5% CO_2_ incubator.

### 2.2 ASC cataract mouse model

All animal experiments were approved by the Animal Ethics Committee of Kunming Medical University. The mouse model of injury‐induced ASC was established as described previously.^26^ Before the surgical procedure, the mice (body weight [BW] = 30‐50 g, n = 6, male) were given a single intraperitoneal injection of pentobarbital sodium (70 mg/kg). The pupil was dilated with tropicamide eye drops. A small incision was then made in the central anterior capsule of the lens in the right eye of the mouse with a 0.26‐mm thick needle blade. The damage depth was approximately 280 μm, which is approximately 1/4 of the partial length of the needle. Animals were allowed to heal for 5, 7 and 14 days before being sacrificed. The lens was removed and paraffin‐embedded, and coronal sections were then taken for immunofluorescence staining haematoxylin‐eosin (HE) and Masson staining.

### 2.3 Haematoxylin‐eosin staining and Masson staining

Paraffin sections were stained with haematoxylin solution (Solarbio, Beijing, China, No.: G1120) for 5 minutes, dipped in a hydrochloric acid‐alcohol solution for 5 seconds and washed with distilled water. Then, slices were stained with eosin solution (Solarbio, No.: G1120) for 3 minutes. Next, sections were dehydrated in a graded alcohol series and cleared in xylene. Finally, sections were observed and imaged under an optical microscope (Olympus).

Masson staining was performed according to the manufacturer's protocol (Solarbio, No.: G1340). Slices were dewaxed conventionally in water, stained with Weigert's iron haematoxylin for 5‐10 minutes, differentiated in a hydrochloric acid‐alcohol solution for 10 seconds and washed with distilled water. After being stained with Biebrich scarlet‐acid fuchsin solution for 5‐10 minutes, sections were washed with 2% aqueous glacial acetic acid solution for 1 minute and were then treated with an aqueous solution of phosphomolybdic acid for 1‐2 minutes. Sections were then stained directly with aniline blue solution for 1‐2 minutes and washed again with acid solution. A 95% ethanol solution was used for dehydration, and slides were sealed with neutral resin. Slides were then observed and imaged under a microscope (Olympus).

### 2.4 Silencing of CNPase with small interfering RNA (siRNA)

To knock down CNPase, either non‐silencing siRNA or three kinds of CNPase‐specific siRNA duplexes purchased from GenePharma were transiently transfected using Lipofectamine 2000 (Thermo Fisher, No.: 11668019) according to the manufacturer's protocol. Briefly, SRA 01/04 cells (1.5 × 10^6^/well) were seeded into 6‐well plates. After 24 hours, at approximately 70% confluency, cells were transfected with negative control siRNA or CNPase‐specific siRNA and incubated at 37°C for 12 hours. Then, the medium was replaced with normal medium and cultured for another 48 hours. Western blot analysis and quantitative real‐time PCR (qRT‐PCR) were performed to monitor transfection efficiency. Transfected cells were further treated with TGF‐β2 (15 ng/mL; R&D Systems, No.: 302‐B2‐010) for different durations (3‐48 hours). The treated cells were used for the next series of experimental analyses.

### 2.5 Lentivirus‐mediated overexpression of CNPase

Lentivirus overexpressing CNPase was purchased from GenePharma. Prior to transfection, SRA 01/04 cells were inoculated in 6‐well plates, at 70%‐80% confluence, the cells were infected with lentivirus. After 48 hours, the infected cells were supplemented with fresh normal medium. Cells with stable CNPase overexpression were obtained by puromycin selection. Transfected cells were further treated with 10 μmol/L DAPT (a specific inhibitor of Notch receptor cleavage, Sigma‐Aldrich) to explore the relationship between CNPase and the Notch signalling pathway.

### 2.6 Western blot analysis

After the desired incubation, SRA 01/04 cells were washed three times with phosphate‐buffered saline (PBS). After this, ice‐cold radioimmunoprecipitation assay (RIPA, Solarbio, No.: R0010) lysis buffer containing 1% phenylmethylsulfonyl fluoride (PMSF, Solarbio, No.: P0100) was added to the cells for total cellular protein extraction. Total cellular protein contents were determined by the BCA method. After mixing with 2× protein loading buffer (Tiangen, No.: RT209), samples were incubated at 95°C for 15 minutes. Equal amounts of protein were loaded onto a 10% SDS‐PAGE gel and transferred to polyvinylidene difluoride (PVDF) membranes (Millipore, No.: IPVH00010). Membranes were blocked in Tris‐buffered saline containing Tween 20 (TBST) and 5% non‐fat dry milk for 3 hours at room temperature and then incubated with the indicated primary antibody (1:1,000 dilution) at 4°C overnight. Following five rinses (10 min/time) with TBST, membranes were incubated with the appropriate horseradish peroxidase (HRP)‐conjugated goat anti‐mouse IgG (Affinity Biosciences, No.: S0002) or HRP‐conjugated goat anti‐rabbit IgG (Affinity Biosciences, No.: S0001) secondary antibodies (1:2000 dilution) for 1.5 hours at room temperature. After washing with TBST five times, protein expression was assessed by chemiluminescence detection reagents (Millipore, No.: WBKLS0100). The greyscale analysis was performed using Gel‐Pro Analyzer 4.0 (Media Cybernetics). The data presented were repeated in triplicate. The sources of the primary antibodies are shown in Table 1.

**Table 1:** The primary antibodies used for Western blotting and immunofluorescence staining

Antibody	Host	Application	Source	Catalogue number
CNPase	Mouse monoclonal	IF, WB	Millipore	No:MAB326
α‐SMA	Mouse monoclonal	WB	Cell Signaling Technology	No:19 245
Vimentin	Rabbit monoclonal	IF, WB	abcam	No:ab92547
Fibronectin	Rabbit monoclonal	WB	abcam	No:ab45688
Collagen Ⅰ	Rabbit polyclonal	IF, WB	abcam	No:ab34710
Collagen Ⅳ	Rabbit polyclonal	IF, WB	abcam	No:ab6586
Slug	Rabbit monoclonal	WB	Cell Signaling Technology	No:9585
E‐Cadherin	Rabbit monoclonal	IF, WB	Cell Signaling Technology	No:3195
Notch‐1	Rabbit monoclonal	IF, WB	Cell Signaling Technology	No:3608
NICD	Rabbit polyclonal	IF, WB	abcam	No:ab8925
RBP‐Jκ	Rabbit monoclonal	IF, WB	Cell Signaling Technology	No:5313
Hes‐1	Rabbit monoclonal	IF, WB	Cell Signaling Technology	No:11 988
GAPDH	Mouse monoclonal	WB	Cell Signaling Technology	No:5174
β‐actin	Mouse monoclonal	WB	Cell Signaling Technology	No:4970

Abbreviations: IF, Immunofluorescence; WB, Western blotting.

### 2.7 Immunofluorescence staining

For this experiment, SRA 01/04 cells grown on sterile glass coverslips were treated with 15 ng/mL TGF‐β2 for 12 hours. Following this, the cells were fixed in 4% paraformaldehyde, permeabilized in 0.1% Triton X‐100 and blocked in 5% bovine serum albumin (BSA). Then, the cells were incubated with primary antibodies (1:200 dilution) at 4°C overnight and then incubated with the secondary antibody (1:2000) for 1 hour in the dark at room temperature. The sources of the primary antibodies are given in Table 1. The secondary antibodies used were as follows: Donkey anti‐Mouse IgG Highly Cross‐Adsorbed Secondary Antibody (Thermo Fisher Scientific, No.: A10036) and Donkey anti‐Rabbit IgG Highly Cross‐Adsorbed Secondary Antibody (Thermo Fisher Scientific, No.: A10040). Cell nuclei were stained with Fluoroshield containing 4,6‐diamidino‐2‐phenylindole dihydrochloride (DAPI, Sigma, No.: F6057‐20ML) and were then mounted. Protein expression in cells was detected with a confocal microscope (Leica TCS SP5), and images were acquired accordingly. LAS‐AF‐Lite_2.6.0 was used for quantifying immunofluorescence labelling.

For paraffin‐embedded slides, sections were deparaffinized and rehydrated according to standard procedures. Antigen was retrieved by heating for 10 minutes in 0.01 mol/L sodium citrate buffer in a pressure cooker. After treatment as described above, immunofluorescence staining was performed to observe changes in the expression of vimentin and CNPase in the ASC mouse model. Fluorescence labelling was visualized and quantified with the tools described above.

### 2.8 qRT‐PCR

Total RNA was isolated from transfected SRA 01/04 cells using RNAiso Plus (TaKaRa, No.: 9109), according to the manufacturer's protocol. The concentration of RNA was determined using a MaestroNano spectrophotometer (MN‐913A, Maestrogen). Total RNA (2 μg) was used as a template to synthesize cDNA using a PrimeScript RT Reagent Kit (Takara, No.: RR047A). For real‐time PCR, TB Green Premix Ex Taq II (TaKaRa, No.: RR820A) was used to amplify target genes, and the data were collected using an Agilent Technologies Stratagene (Mx3005P). Glyceraldehyde 3‐phosphate dehydrogenase (GAPDH) served as an internal control. All the primers were synthesized by Tsingke. The primer sequences are shown in Table 2.

**Table 2:** The primer sequences used for quantitative real‐time PCR

Name	Sequence
CNPase Forward	GCCGCCGGGACATCA
CNPase Reverse	ACTGGTCGGCCATTTCAAAG
GAPDH Forward	GACCTGACCTGCCGTCTAGAAA
GAPDH Reverse	CCTGCTTCACCACCTTCTTGA

### 2.9 Transwell migration assay

For the migration assay, 24‐well transwell chambers (Corning Incorporated, No.: 3422) with an 8 μm pore polycarbonate membrane were used. On the second day after transfection, 2 × 10^4^ cells per well were seeded into the upper chamber and supplemented with DMEM without serum, while the medium in the lower chamber contained 10% FBS as a source of chemoattractants. After incubation for 48 hours, the cells were treated with TGF‐β2 (15 ng/mL). Twenty‐four hours later, the membranes were washed in PBS at room temperature, fixed in 4% formaldehyde and then stained with crystal violet. Migrated cells in five random visual fields were imaged via microscopy (Olympus) and enumerated with ImageJ software. Each trial consisted of three individual wells, and the transwell migration assay was repeated three times.

### 2.10 Wound healing assay

Transfected SRA 01/04 cells were seeded into 12‐well plates and cultured until 100% confluency. The cells were scratched using a sterile plastic pipette tip (10 μL). Following this, the cells were washed with PBS to remove debris. Then, fresh medium was added to each well with or without TGF‐β2 (15 ng/mL). After incubation at 37°C for 24 hours, the wounded areas were imaged. The wounded areas were measured by ImageJ. The data were quantified as follows: cell migration percentage (%) = (new scratch area—original scratch area)/original scratch area × 100%. The wound healing assay was repeated in triplicate.

### 2.11 Cell proliferation assay

The real‐time cell analysis (RTCA) iCELLigence system (CA 92121, ACEA Biosciences, Inc) based on electrical impedance measurements was used to detect cell proliferation. The RTCA iCELLigence system was placed in the incubator at 37°C and with 5% CO_2_. First, 200 μL of DMEM was added to the 8‐well E‐plates, and the background reading was determined. Then, 300 μL of the cell suspension was added to each well, and the cell density was 3 × 10^4^/well. After 6 hours of culture, TGF‐β2 was added, and the cell proliferation rate was recorded every hour. The data are expressed as a normalized unit index.

### 2.12 EdU staining assay

To determine the effect of CNPase on cell proliferation, a Cell‐Light EdU Apollo 488 In Vitro Kit (RiboBio, No.: C10337) was used according to the manufacturer's protocol. In brief, SRA 01/04 cells were seeded into 96‐well plates. Forty‐eight hours after transfection, reagent A was diluted with complete medium at a ratio of 1000:1; the cells were then labelled with EdU (100 μL/well) for 2 hours. The cells were washed with PBS and fixed with 50 μL/well of fixative (PBS containing 4% paraformaldehyde) at room temperature for 30 minutes. Next, the cells were incubated with 2 mg/mL glycine (50 μL/well) for 5 minutes. After washing with PBS (100 μL/well) for 5 minutes, the cells were incubated with 100 μL/well of penetrant (PBS containing 0.5% Triton X‐100) for another 10 minutes and then washed once with PBS (5 minutes). Subsequently, the cells were incubated with 1× Apollo staining solution (100 μL/well) and 1× Hoechst 33342 reaction solution in the dark for 30 minutes. After an additional 10 minutes of treatment with the penetrant, the cells were washed twice with methanol. Signals were detected using a confocal microscope (Olympus). Images of positively stained cells were captured and counted by ImageJ.

### 2.13 Statistical analysis

Statistical Product and Service Solutions 16.0 software (IBM SPSS) was used for statistical analysis. All measurement data are presented as the mean ± standard deviation (SD) from at least three independent experiments. Student's *t* test was used for two‐sample analysis, and one‐way analysis of variance (ANOVA) was applied to compare the mean among three or more groups. A *P* value <.05 was considered to indicate a statistically significant difference.

## 3 RESULTS

### 3.1 Upregulation of CNPase in lens epithelial cells of the ASC mouse model

**Figure 1:**
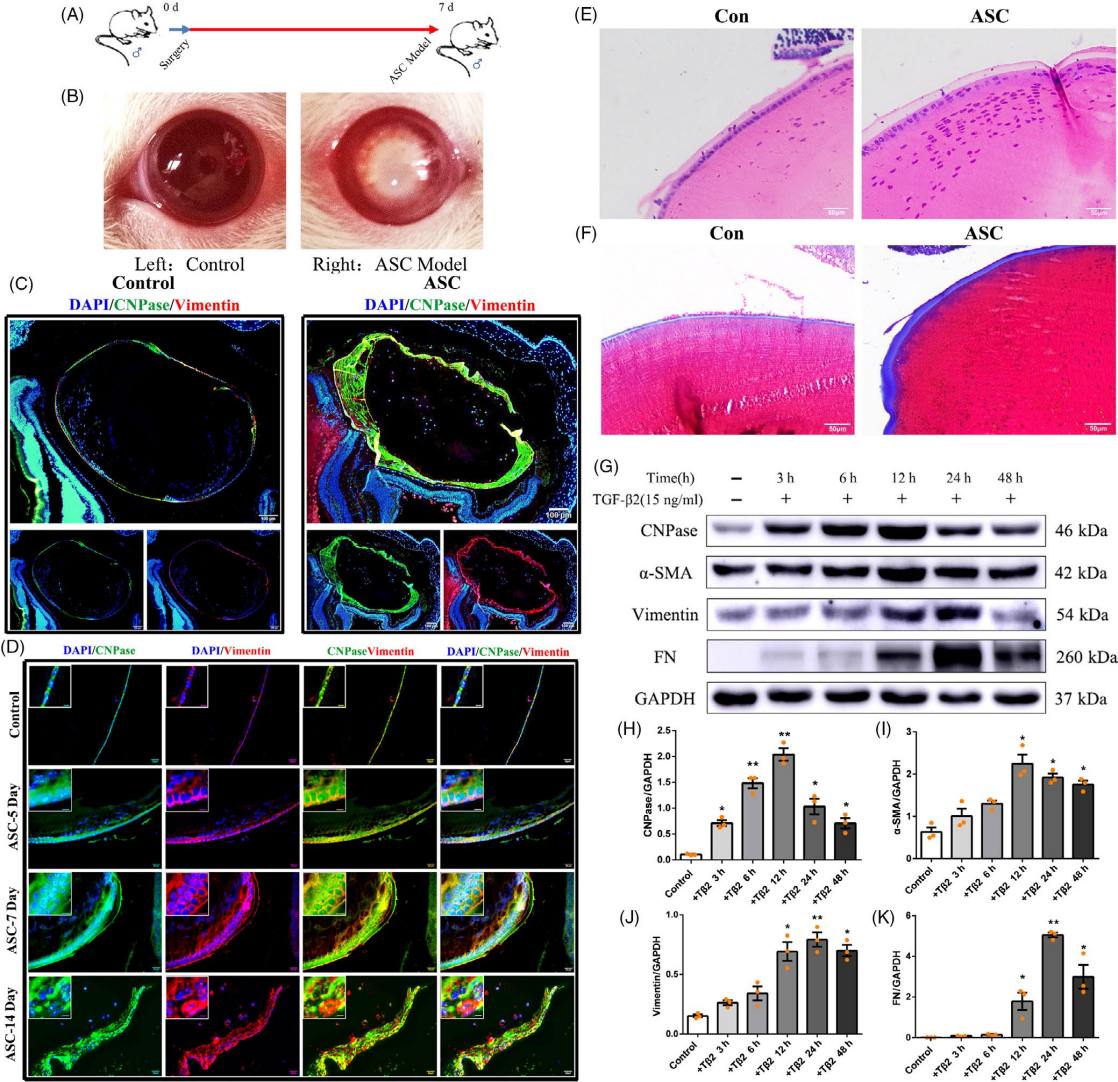
CNPase was upregulated in lens epithelial cells in an injury‐induced ASC mouse model and TGF‐β2‐induced EMT. A, B, Generation of the injury‐induced ASC mouse model and eyeball appearance of the injury‐induced ASC mouse model. Left: healthy control, Right: ASC model. C, D, Haematoxylin‐eosin (HE) and Masson staining. HE staining showed that lenses developed multilayered lens epithelial cell beneath the anterior lens capsules compared with the control left eyes. Masson staining indicated lens fibrosis in ASC mice. E, F, Immunofluorescence showed moderate expression of CNPase (green) in normal lens epithelial cells. CNPase expression was noticeably increased in lens epithelial cells in ASC. Moreover, the expression of the EMT‐related protein vimentin (red) was detected (see “inset”). Scale bars (left) = 100 μm, scale bars (right) = 20 μm. (G‐K) Expression of CNPase, α‐SMA, vimentin and FN in lens epithelial cells treated with TGF‐β2 at different times. **P value <.01, *P value <.05

To explore the biological functions of CNPase in the lens, we observed the expression of CNPase in healthy lenses and changes in its expression in ASC (Figure 1A). On days 5, 7 and 14, lenses developed marked multilayered lens epithelial cell opacity beneath the anterior lens capsule compared with the control left eyes (Figure 1B and 1). Masson staining indicated significant fibrosis in the lens capsules of mice with ASC (Figure 1D). Immunofluorescence labelling showed that CNPase was virtually undetected in the mouse lens fibres but was moderately expressed in epithelial cells of untreated lenses in vivo (Figure 1E). We next examined the changes in CNPase expression in the mouse model of ASC induced by injury. Interestingly, compared with the normal left lenses, the lens epithelial cells in the mouse model exhibited augmented CNPase immunofluorescence (Figure 1F).

### 3.2 CNPase may be involved in the EMT process of lens epithelial cells

EMT is a crucial pathophysiological mechanism of ASC. Because the expression of CNPase in ASC was altered, we next explored the changes in the expression of EMT markers concurrent with the upregulation of CNPase. Enhanced expression of vimentin, a key marker of EMT, was detected in the lens epithelial cells of ASC mice compared with that in the sham lenses (Figure 1F).

TGF‐β2 has been reported to induce EMT of epithelial cells. To further investigate the expression of CNPase during EMT induced by TGF‐β2 in SRA 01/04 cells, lens epithelial cells were treated with TGF‐β2 (15 ng/mL). Changes in the expression of CNPase were then evaluated by Western blot analysis. When treated with TGF‐β2 for 3 hours, CNPase expression began to increase significantly compared with that of the untreated group. At 6 hours and 12 hours, CNPase expression increased gradually in a time‐dependent manner. At 24 hours and beyond, CNPase expression remained at a steadily high level (Figure 1G and H). The expression levels of EMT marker proteins, namely, vimentin, α‐SMA and FN, were increased significantly in SRA 01/04 cells (Figure 1G and I‐K). Overall, the expression of CNPase was positively correlated with that of vimentin, thus supporting the idea that CNPase may mediate EMT to participate in the occurrence of ASC in vivo and in vitro.

### 3.3 Silencing of CNPase inhibits the proliferation of lens epithelial cells

To further investigate the role of CNPase in the EMT process of lens epithelial cells, an siRNA was used to knockdown the *CNPase* gene in SRA 01/04 cells. SRA 01/04 cells were transfected with a control siRNA or three different kinds of CNPase siRNAs (#1257, #859 and #942). All siRNAs were labelled with fluorescent fragments. The results of fluorescence microscopy showed that all four kinds of siRNAs were successfully transfected into SRA 01/04 cells (Figure S1A). The cDNA of lens epithelial cells treated with CNPase siRNAs was extracted, and the knockdown efficiency of CNPase was determined by qRT‐PCR. The knockdown efficiencies of the CNPase siRNAs with structures #942 and #859 were high; after 24 hours of transfection, the knockdown efficiencies were approximately 75% and 69%, respectively, compared with control siRNA‐transfected cells (Figure S1B). At 48 hours after transfection with siRNA construct #942, Western blot analysis showed that the expression level of CNPase protein was decreased by as much as 88.5% (Figure S1C). Therefore, we used the CNPase siRNA with structure #942 (siCNP‐942) for subsequent functional analysis.

**Figure 2:**
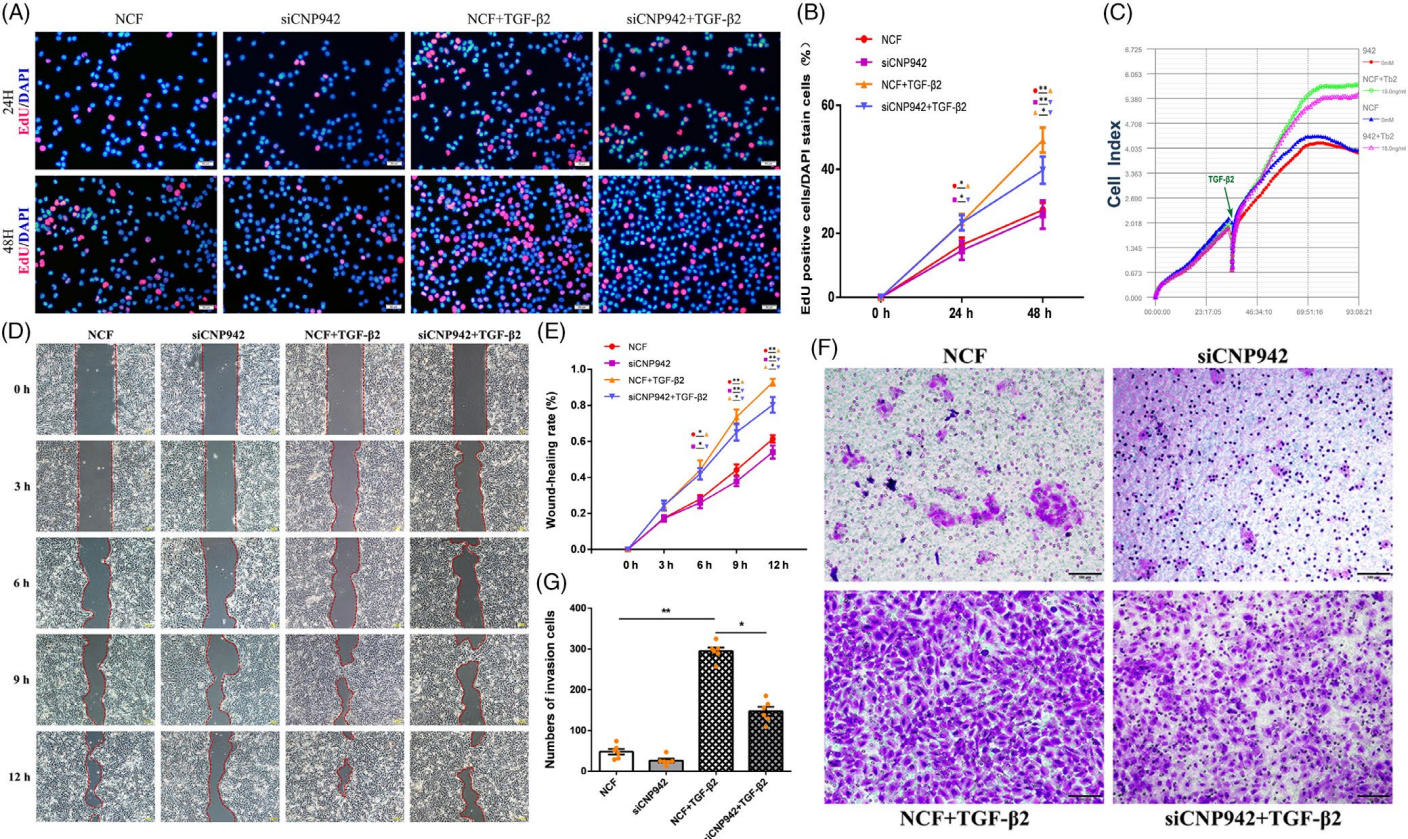
Silencing of CNPase downregulated the proliferation and migration of lens epithelial cells. A, CNPase gene silencing reduced the percentage of SRA 01/04 cells in S phase following treatment with TGF‐β2. B, The percentage of proliferating cells in CNPase siRNA‐transfected cells was significantly lower than that in control siRNA‐transfected cells. C, The iCELLigence system showed the decrease in the proliferation of SRA 01/04 cells with CNPase knockdown. D‐E, Wound healing assays showed that CNPase knockdown significantly inhibited the migration of lens epithelial cells. F‐G, Transwell assays showed that TGF‐β2 increased the number of migrated cells. In contrast, CNPase silencing decreased the number of migrated lens epithelial cells. **P value <.01, *P value <.05. Scale bars = 100 μm

The proliferation ability of SRA 01/04 cells with or without CNPase knockdown was observed (Figure 2A‐C). CNPase gene silencing reduced the percentage of SRA 01/04 cells in S phase following treatment with TGF‐β2. The percentage of proliferating cells in siCNP‐942‐transfected cells was significantly lower than that in control siRNA‐transfected cells. Similarly, the percentage of CNPase siRNA‐transfected cells in S phase (23%) was lower than that in lens epithelial cells after TGF‐β2 treatment (39%) (Figure 2A and 2).

### 3.4 Knockdown of CNPase downregulates TGF‐β2‐induced EMT in lens epithelial cells

To further understand the biological function of CNPase in EMT, we transfected SRA 01/04 cells with siCNP‐942. The migration and TGF‐β2‐induced EMT of lens epithelial cells were detected by wound healing and transwell migration assays. Wound healing experiments showed that the knockdown of CNPase significantly inhibited the migration of lens epithelial cells. Next, an in vitro model of EMT was established using TGF‐β2_,_ which markedly promoted cell migration (Figure 2D and 2). Compared with SRA 01/04 cells treated with TGF‐β2, lens epithelial cells treated with siCNP‐942 exhibited inhibited migration. We further detected the EMT characteristics of lens epithelial cells via a transwell assay. As shown in Figure 2, TGF‐β2 increased the number of migrated cells. In contrast, silencing of CNPase decreased the number of migrated lens epithelial cells (Figure 2F and G).

**Figure 3:**
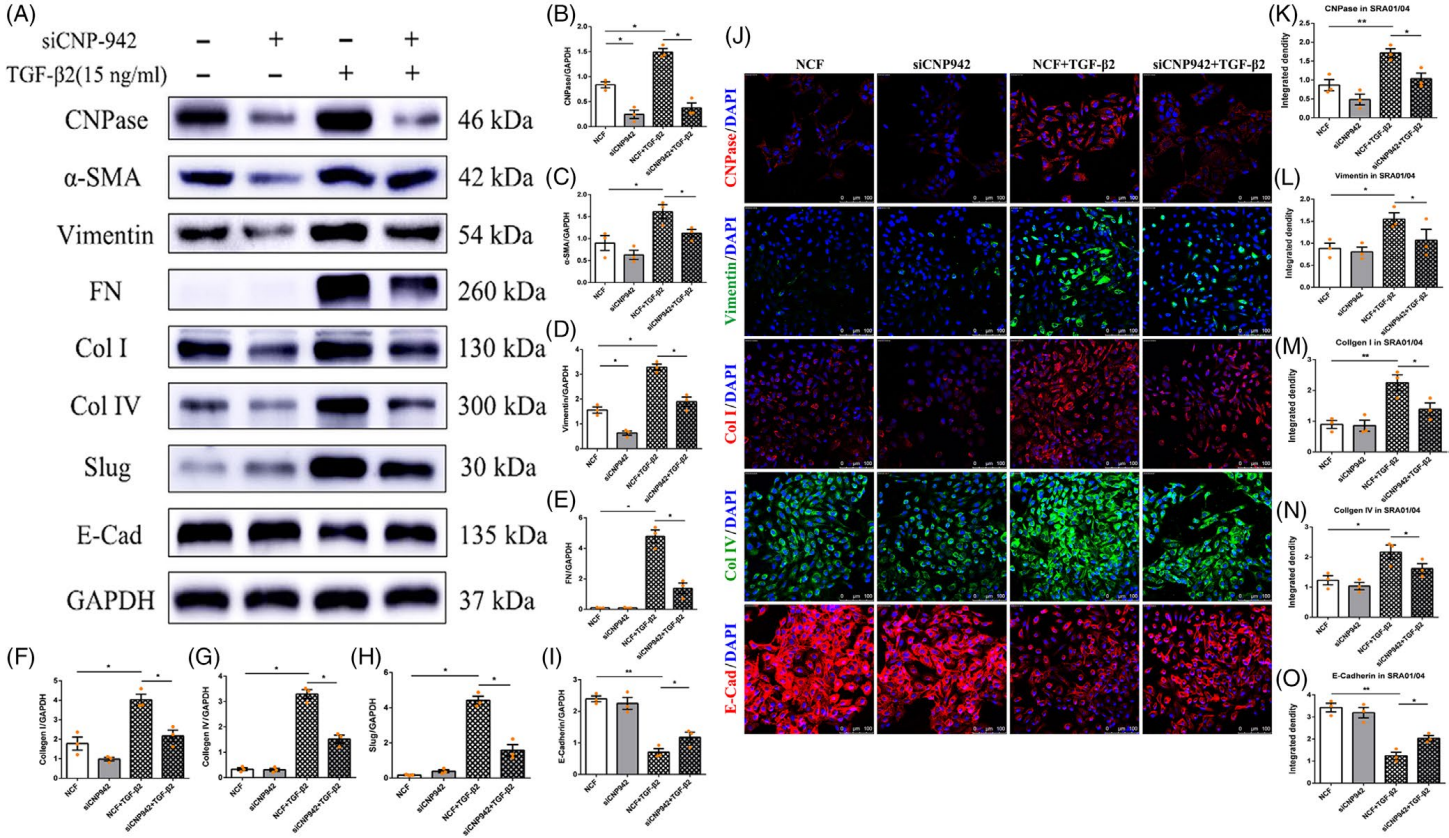
CNPase knockdown inhibited the expression of EMT markers in lens epithelial cells. A‐I, Western blot analysis showed that CNPase silencing reduced the TGF‐β2‐induced increase in vimentin, α‐SMA, FN, Col I, Col IV and Slug (Snail2) expression in lens epithelial cells. However, CNPase knockdown upregulated the expression of E‐cadherin. J‐O, Immunofluorescence suggested that silencing of CNPase downregulated the TGF‐β2‐induced increase in vimentin, Col I and Col IV expression, while E‐cadherin expression was upregulated. **P value <.01, *P value <.05. Scale bars = 100 μm

Additionally, the results of Western blotting suggested that TGF‐β2 induced the increased expression of vimentin, α‐SMA, FN, Col I, collagen type IV (Col IV) and the EMT transcription factor Slug (Snail2) in lens epithelial cells. In contrast, silencing of CNPase reduced the TGF‐β2‐induced increase in vimentin, α‐SMA, FN, Col I, Col IV and Slug (Figure 3A‐H). Moreover, the change in the expression of E‐cadherin, a protein marker of epithelial cells, was opposite that of the aforementioned proteins (Figure 3A and I). Immunofluorescence staining further confirmed the above results of Western blotting (Figure 3J‐O). In summary, these results suggest that CNPase is involved in the regulation of the EMT phenotype in lens epithelial cells.

### 3.5 The Notch signalling pathway participates in EMT induced by CNPase

**Figure 4:**
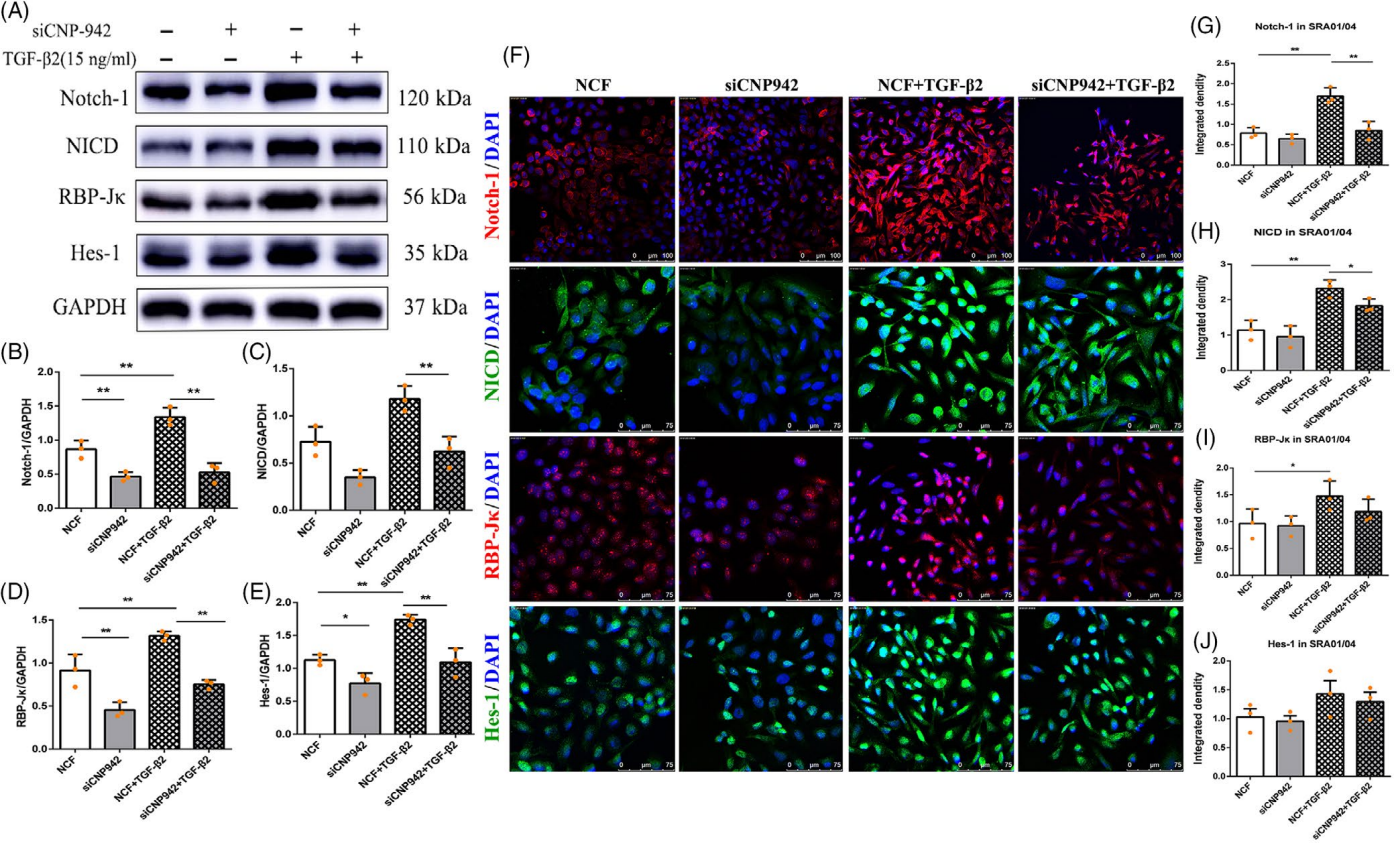
The Notch signalling pathway participates in EMT induced by CNPase. A‐E, TGF‐β2 significantly increased the expression of the Notch pathway receptor Notch‐1 and concurrently upregulated the expression of its downstream genes, RBP‐Jκ and Hes‐1. However, activation of Notch signalling induced by TGF‐β2 was inhibited by CNPase siRNA. F‐J, Immunofluorescence showed that the expression of Notch‐1 and NICD was decreased following CNPase siRNA treatment. **P value <.01, *P value <.05. Scale bars = 100 μm

In light of the critical role of the Notch signalling pathway in the pathogenesis of EMT, we next explored whether CNPase was involved in TGF‐β2‐induced EMT by regulating the Notch pathway. As shown in Figure 4, TGF‐β2 markedly induced the expression of the Notch pathway receptor Notch‐1 and upregulated the expression of its downstream genes, RBP‐Jκ and Hes‐1. However, activation of Notch signalling induced by TGF‐β2 was inhibited by CNPase siRNA (Figure 4A‐E). These results were further confirmed by an immunofluorescence assay. TGF‐β2 significantly increased the integrated density of Notch‐1, NICD, RBP‐Jκ, and Hes‐1 in SRA 01/04 cells compared with control siRNA‐treated cells. However, the expression of Notch‐1 and NICD decreased following siCNP‐942 treatment (Figure 4F‐J).

**Figure 5:**
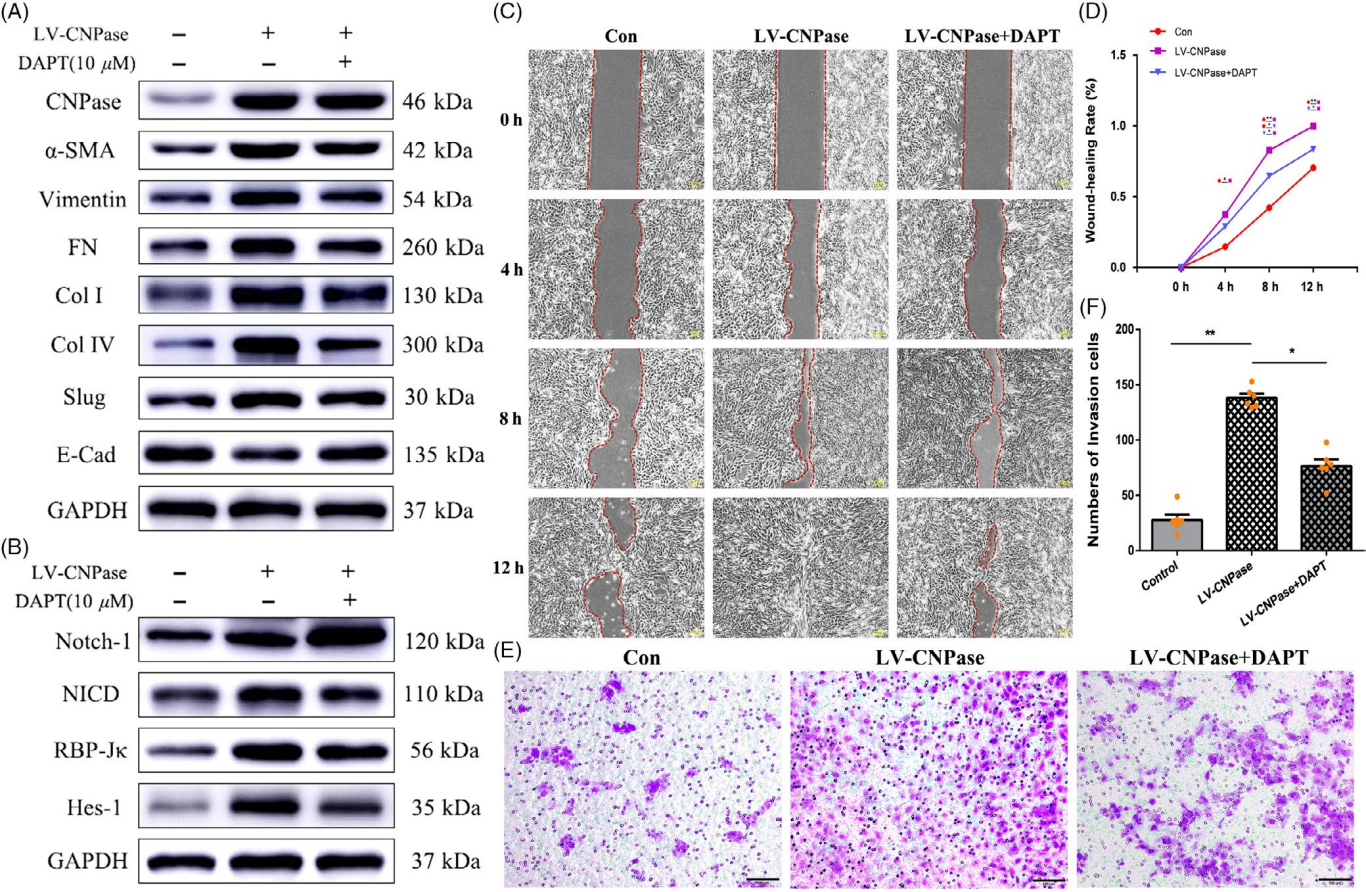
Blockade of the Notch pathway with DAPT prevents CNPase‐induced EMT in vitro. A, Western blotting showed that LV‐CNPase was successfully transfected into SRA 01/04 cells. Western blotting showed that DAPT inhibited the increase in vimentin, α‐SMA and FN expression induced by CNPase overexpression, while the expression of E‐cadherin was downregulated. B, CNPase upregulated the expression of the Notch pathway receptor Notch‐1 and its downstream genes, RBP‐Jκ and Hes‐1. DAPT inhibited the activation of Notch signalling induced by CNPase overexpression. C, D, Wound healing assays showed that LV‐CNPase significantly promoted the migration of lens epithelial cells. DAPT can partially inhibit the migration of LV‐CNPase transfected lens epithelial cells. E, F, Transwell assays showed that LV‐CNPase increased the number of migrated cells. In contrast, DAPT reduced the migration of LV‐CNPase‐transfected lens epithelial cells. **P value <.01, *P value <.05. Scale bars = 100 μm

To further determine whether CNPase regulates the Notch signalling pathway, we constructed lentivirus overexpressing CNPase (LV‐CNPase) and transfected this lentivirus into SRA 01/04 cells. Western blotting showed that CNPase expression was significantly increased after transfection of LV‐CNPase (Figure 5A). Therefore, we used SRA 01/04 cells stably transfected with LV‐CNPase for the follow‐up experiment.

SRA 01/04 cells were treated with DAPT, and the key factors in the Notch pathway were assessed. The expression levels of Notch‐1, NICD, RBP‐Jκ and Hes‐1 in LV‐CNPase SRA 01/04 cells were significantly higher than those in cells treated with empty vector. After treatment with DAPT, the expression of Notch‐1, NICD, RBP‐Jκ and Hes‐1 in LV‐CNPase SRA 01/04 cells was downregulated (Figure 5B). Then, we further detected the EMT characteristics of lens epithelial cells in vitro. Compared with SRA 01/04 cells treated with empty vector, lens epithelial cells with CNPase overexpression exhibited enhanced migration (Figure 5C‐F). As shown in Figure 5, along with the above changes, the expression of key EMT proteins also changed accordingly. The expression of vimentin, Col IV and α‐SMA in LV‐CNPase‐transfected SRA 01/04 cells was increased compared with that in cells transfected with empty vector. In addition, DAPT downregulated the expression of vimentin, Col IV and α‐SMA in LV‐CNPase SRA 01/04 cells (Figure 5A). Taken together, these results suggest that activation of the Notch pathway is involved in EMT induced by TGF‐β2. The mechanism by which CNPase promotes EMT may act via direct targeting of the Notch signalling pathway.

## 4 DISCUSSION

Pathological EMT has been considered the main driver of ASC. Clinical cataract surgery and intraocular lens design highlight the importance of lens epithelial cell EMT in the pathogenesis of ASC.^27^ Multiple signalling pathways, including the Notch and Wnt signalling pathways, have historically been considered to be involved in the EMT process in ASC.^28^ However, the specific mechanism that triggers and maintains EMT has remained obscure. In this study, we focused on the roles of CNPase in the process of regulating the occurrence of EMT in lens epithelial cells. The present results showed that CNPase is constitutively expressed in lens epithelial cells and that it is upregulated in ASC. The functional interactions between CNPase and the transcriptional regulation of EMT‐related genes were then explored and confirmed. The present results unequivocally demonstrate that CNPase plays a pivotal role in the mesenchymal targeting and regulation of EMT induced by TGF‐β2, which may provide evidence for the participation of CNPase in the pathogenesis of ASC.

CNPase exists in many eukaryotes and is expressed at a low level in many mammalian tissues. Previous studies have reported that CNPase is expressed in the heart, spleen and kidney and is highly expressed in oligodendrocytes; indeed, it is one of the major marker molecules in the process of myelin formation in oligodendrocytes.^29^, ^30^ As a specific marker of oligodendrocytes and an important component of myelin in the central nervous system, CNPase is of special significance in the diagnosis of demyelinating diseases of the central nervous system, such as multiple sclerosis.^29^, ^31^ However, studies on the expression of CNPase in healthy human lens epithelial cells have remained elusive, especially in ASC lens epithelial cells. In this study, we found, for the first time, that CNPase is expressed in normal lens epithelial cells. More importantly, CNPase expression was upregulated and maintained at a high level during the development of ASC. Previous studies verified that in the process of ASC, EMT of lens epithelial cells inevitably occurred, leading to the production of extracellular matrix and the transformation of epithelial cells into cells with a mesenchymal phenotype.^27^, ^32^ We found that the expression of vimentin, a key protein involved in EMT and mesenchymal derived cells, was increased in lens epithelial cells of the ASC mouse model and positively correlated with CNPase. These results reveal that CNPase may be involved in EMT of lens epithelial cells and participate in the development of ASC.

The physiological substrates and specific biological roles of CNPase in mammalian cells need to be fully elucidated. Several studies have suggested that CNPase is associated with apoptosis and inflammation.^33^ Therefore, whether CNPase participates in EMT requires in‐depth verification. The treatment of lens epithelial cells with TGF‐β2 is a common cell model for EMT and fibrosis. To further confirm that CNPase is involved in EMT, the present results showed that the expression of CNPase was increased in TGF‐β2‐induced lens epithelial cells. This effect was significant; even after 3 hours of TGF treatment in lens epithelial cells, the expression of CNPase was significant and remained high at 72 hours. We note that this change in CNPase expression is consistent with the phenotypic changes in lens epithelial EMT. This study describes the underlying molecular mechanism of EMT, underscoring the key role of CNPase in promoting the activation of EMT‐associated genes.

To further understand the biological functions of CNPase, we investigated the effects of CNPase silencing on the proliferation and migration of lens epithelial cells. The EdU assay showed that CNPase knockdown significantly inhibited the proliferation of lens epithelial cells. Notably, the wound healing and transwell migration assays showed that the migration of lens epithelial cells was significantly decreased after CNPase knockdown. In the process of EMT, downregulation of E‐cadherin and overexpression of vimentin or Slug are considered key factors.^34^ Western blotting and immunofluorescence showed that when the CNPase gene was silenced, E‐cadherin expression was increased, whereas the expression of vimentin, α‐SMA, FN, Col I, Col IV and Slug was decreased. In summary, the results showed that CNPase may play a negative role in cell migration and the EMT process by regulating E‐cadherin and Slug.

**Figure 6:**
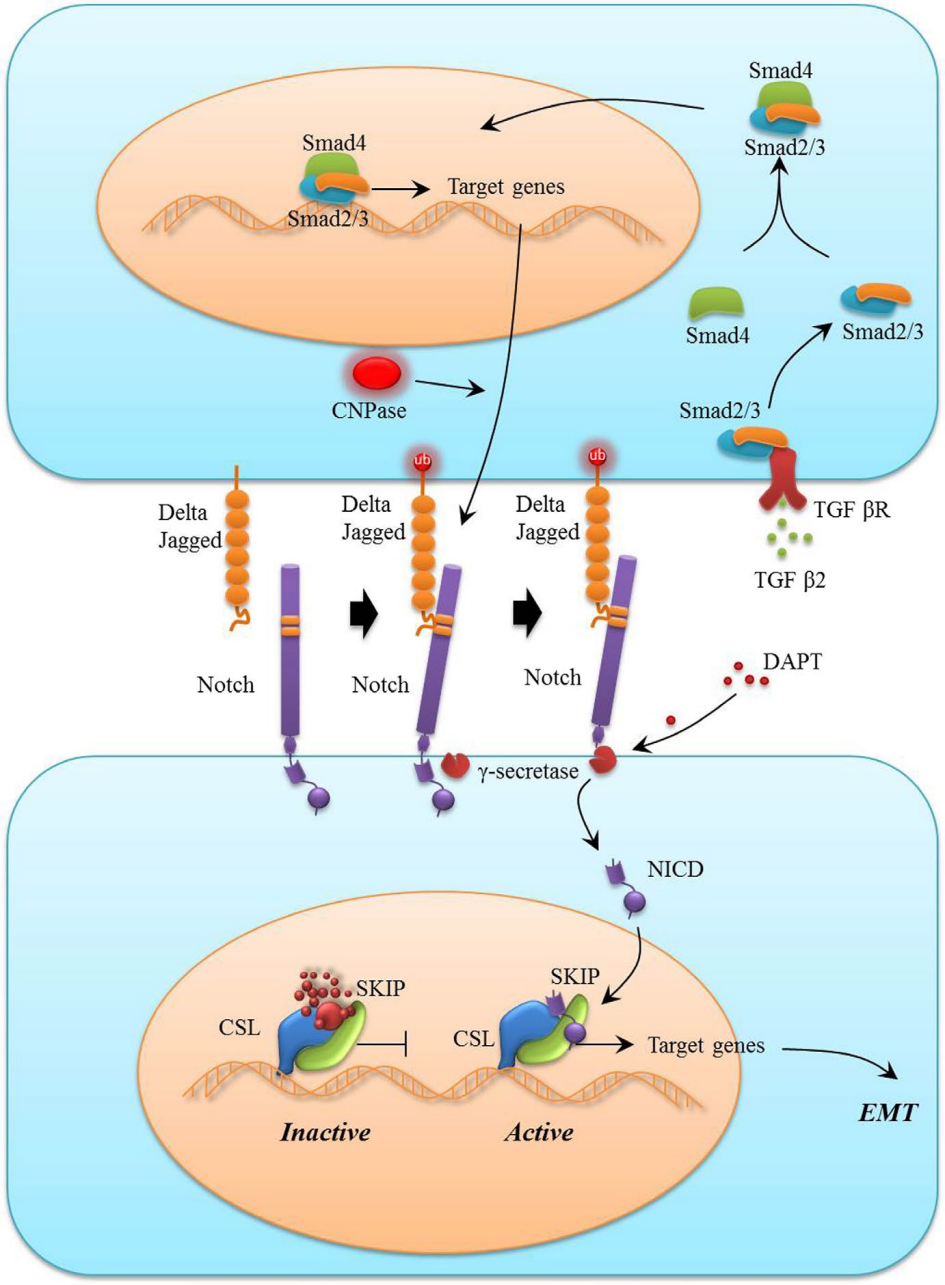
Schematic showing the role of CNPase/Notch signalling pathway in modulating EMT and fibrosis. During the EMT process, CNPase activates EMT through the canonical Notch signalling pathway. After ligand binding, the Notch receptor is cleaved by γ‐secretase, and NICD is released and subsequently translocates into the nucleus to regulate the expression of downstream target genes, leading to EMT and fibrosis. During this process, CNPase can target the regulation of Notch‐1 to upregulate Notch signalling pathway activity, thus promoting EMT and fibrosis. Moreover, DAPT, a specific inhibitor of the Notch pathway, can reverse EMT and fibrosis

Notch signalling has been reported to play a crucial role in EMT in various fibrotic diseases and cancer metastasis, and its function in EMT of lens epithelial cells has been confirmed.^28^ However, whether CNPase activates Notch signalling and participates in the development of EMT has not been reported. We found that CNPase positively regulated the expression of Notch‐1 and its downstream key factors. The Notch signalling pathway has been considered the key regulatory pathway for embryonic development, tumour invasion and fibrotic diseases. Chen et al showed that Notch signalling was activated in the TGF‐β2‐induced EMT of lens epithelial cells, and blocking Notch signalling reversed EMT and lens fibrosis.^28^ Consistent with these results, our results suggest that Notch‐1, RBP‐Jκ and Hes‐1, members of the Notch signalling pathway, were activated during TGF‐β2‐induced EMT in lens epithelial cells. Notably, this effect was dampened by CNPase silencing. To further observe whether CNPase is involved in the Notch signalling pathway, we used DAPT, a γ‐secretase inhibitor, to block Notch signalling pathway activity and observe the EMT characteristics of CNPase overexpression. DAPT obviously inhibited the induction of the Notch signalling pathway by CNPase; downregulated the expression of vimentin, α‐SMA and Col IV in lens epithelial cells; and alleviated the associated EMT phenotype. Hence, it is reasonable to conclude that CNPase, at least in part, regulates EMT in lens epithelial cells in a manner mediated by regulation of the Notch signalling pathway (Figure 6).

In conclusion, an ASC mouse model was used to show for the first time that CNPase is highly expressed and related to EMT marker protein expression in lens epithelial cells. Very strikingly, CNPase promotes the proliferation, migration and EMT of lens epithelial cells by targeting Notch signalling in vitro. Therefore, clarifying the regulatory relationship between CNPase and the occurrence of EMT is desirable, as it not only would improve the understanding of ASC pathogenesis but also may suggest the effectiveness of blocking CNPase and the Notch signalling pathway as an effective strategy for preventing and treating ASC.

## CONFLICT OF INTEREST

The authors have no conflicts of interest.

## Data Availability

The data that support the findings of this study are available from the corresponding author upon reasonable request.
