# Supplementary figures and images for: 2',3'‐Cyclic‐nucleotide 3'‐phosphodiesterase contributes to epithelial‐mesenchymal transition of lens epithelial cells through the notch signalling pathway
Source: Cell Prolif. 2019 Oct 16;52(6):e12707. doi: 10.1111/cpr.12707 (PMC6869463; doi:10.1111/cpr.12707)

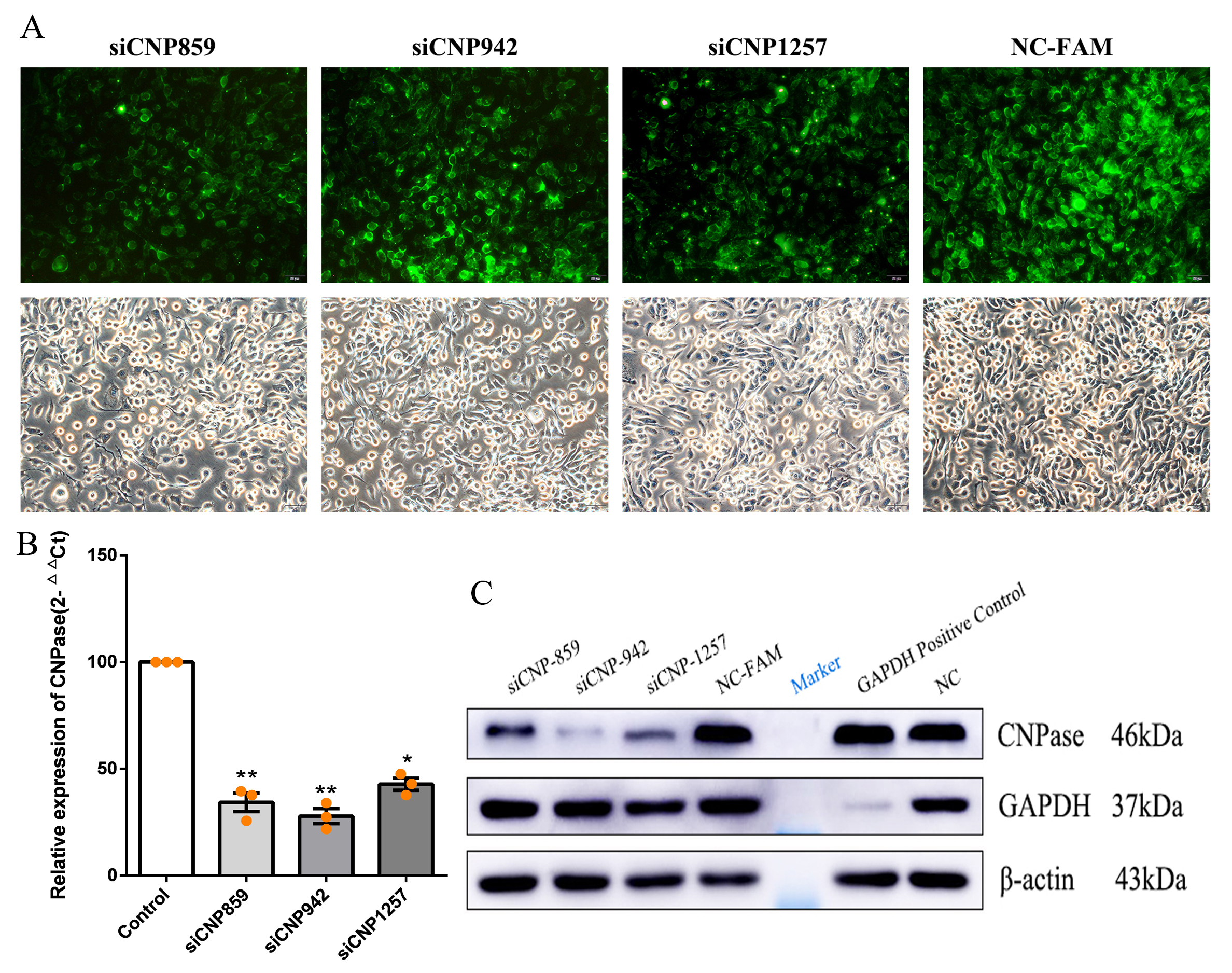

Supplement: Supplementary file 1 [file CPR-52-e12707-s001.jpg]
